# Predicting the evolution of antibiotic resistance

**DOI:** 10.1186/1741-7007-11-14

**Published:** 2013-02-22

**Authors:** Martijn F Schenk, J Arjan GM de Visser

**Affiliations:** 1 Institute for Genetics, University of Cologne, Zulpicher Strasse 47, D-50674, Koln, Germany; 2 Laboratory of Genetics, Wageningen University, Droevendaalsesteeg 1, 6708PB, Wageningen, the Netherlands

## Abstract

Mutations causing antibiotic resistance are often associated with a cost in the absence of antibiotics. Surprisingly, a new study found that bacteria adapting to increased temperature became resistant to rifampicin. By studying the consequences of the involved mutations in different conditions and genetic backgrounds, the authors illustrate how knowledge of two fundamental genetic properties, pleiotropy and epistasis, may help to predict the evolution of antibiotic resistance.

See research article http://www.biomedcentral.com/1471-2148/13/50

## Commentary

The spread of pathogenic bacteria that are resistant to certain antibiotics constitutes a growing threat to public health. One strategy to counter this problem is to minimize the probability that antibiotic resistance arises. To achieve this we need to understand the factors that drive and constrain its evolution [1]. The fitness costs associated with resistance have received considerable attention since they determine the long-term success of resistant bacteria. Such costs may arise from modifications of cellular components with vital functions, such as ribosomes or the cell wall, or from metabolic costs due to the expression of enzymes that break down the antibiotic. These pleiotropic costs would allow resistance to be selected only when bacteria are confronted with antibiotics. Surprisingly, a study published in *BMC Evolutionary Biology *[2] now reports the fixation of mutations causing rifampicin resistance in *Escherichia coli *populations that evolved at high temperature in medium without any antibiotics. By gauging the fitness consequences of these mutations in other environments and in other bacterial strains, the authors show that the beneficial effects of these mutations are highly specific and highlight two factors that are crucial for the evolution of antibiotic resistance: pleiotropy and epistasis.

The study by Rodríguez-Verdugo *et al*. [2] analyses *E. coli *lines from a previously reported large-scale evolution experiment with 114 populations that have evolved for 2,000 generations at an increased temperature of 42.2°C [3]. Genome sequencing of clones from all evolved populations showed that 74 clones contained at least one of 46 unique non-synonymous mutations in the *rpoB *gene [3], which encodes the β-subunit of RNA polymerase and is a known target of rifampicin resistance [4]. Thirteen of the 114 lines did acquire intermediate to very high levels of rifampicin resistance. Interestingly, all 13 resistant lines had a non-synonymous mutation in *rpoB*, and 12 showed one of three mutations at codon position 572, thus showing signatures of parallel evolution - a hallmark of their involvement in adaptation. By looking at the temporal dynamics the researchers showed that these mutations appeared early in the selection, again consistent with their involvement in adaptation. The authors obtained definitive proof that the three mutations at position 572 increase fitness in the evolutionary environment by testing their effect in the ancestral strain, revealing fitness increases of about 20%.

How surprising are these results? Resistance mechanisms are generally thought to be costly since antibiotics target fundamental cellular processes, including the synthesis of mRNA, proteins and cell structures [4]. Fitness costs associated with resistance may arise from modifications in the cellular targets of antibiotics, which prevent binding of the drug but also compromise their cellular role. This is, for instance, the case for resistance to rifampicin and streptomycin, which target the β-subunit of RNA polymerase and ribosomal protein S12, respectively. Costs may also be due to the synthesis of enzymes that break down antibiotics, as is the case for β-lactams, or when porin mutations that interfere with the antibiotic entering the cell also limit the uptake of nutrients, as for penicillin and tetracycline. Many studies have quantified such costs in the absence of the antibiotic under various *in vitro *and *in vivo *conditions [4]. But a 'cost of resistance' was not always observed, and neutral or even beneficial effects in the absence of the drug have previously been observed for resistance to a number of other antibiotics (reviewed in [4]). Complementary to the results of Rodríguez-Verdugo *et al*., a recent study showed that *E. coli *selected in the presence of rifampicin acquired mutations in the *rpoB *gene that were beneficial in minimal medium at increased temperature [5]. However, other than the study by Rodríguez-Verdugo *et al*., these studies lacked genomic information and could not rule out the possibility that secondary mutations have occurred that compensated for the negative pleiotropic effects of the resistance mutations.

## Pleiotropy and epistasis

**Figure 1:**
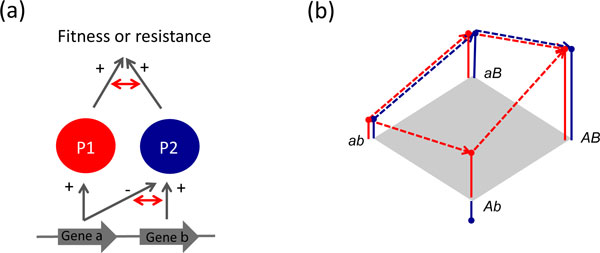
**Pleiotropy and epistasis are key factors in the evolution of antibiotic resistance**. **(a) **Definition of pleiotropy and epistasis. Phenotypes P1 and P2 affect fitness (or antibiotic resistance) and are determined by genes *a *and *b*. Plus/minus signs indicate positive/negative effects. Gene *a *has antagonistic pleiotropic effects on both phenotypes, while gene *b *is not pleiotropic. Epistasis is the dependence of the phenotypic or fitness effect of a gene on the effect of another gene; epistasis at the level of fitness may thus arise from gene interactions determining underlying phenotypes or from interactions among phenotypes in their effect on fitness, or both (opportunity for epistasis indicated by double-headed red arrows). **(b) **Knowledge of pleiotropy and epistasis allows evolutionary predictions. Shown are fitness effects of alleles *A *and *B *in a red and in a blue environment. In both environments, *AB *is the fittest (or most resistant) combination. In the red environment, *A *and *B *are not epistatic and both pathways from *ab *to *AB *(shown by red arrows) are equally likely. However, in the blue environment, alleles *A *and *B *show sign epistasis, leading to the low-fitness combination *Ab*. As a result, one pathway (*ab *> *aB *> *AB*, shown by blue arrows) is much more likely under the influence of natural selection than the other (*ab *> *Ab *> *AB*), making evolution relatively predictable.

Rodríguez-Verdugo *et al*. further tested the effect of the *rpoB *mutations in other bacterial strains, in other growth media and at other temperatures (in the absence of rifampicin), and found that the benefits are specific for the environmental conditions and genetic background used. Their study thus illustrates the crucial role of pleiotropy and epistasis as factors that constrain the evolution of antibiotic resistance. Pleiotropy occurs when a single mutation affects multiple phenotypic traits, and epistasis when mutations at different loci interact in non-additive ways in their effect on a phenotype or on fitness. These features reflect the genetic wiring connecting genotypes and phenotypes (Figure 1a), and can be considered fundamental properties of biological systems. Evolutionary constraints from pleiotropy arise when mutations have positive effects on one phenotype and negative effects on another, thereby making adaptation specific for conditions requiring the first phenotype. An additional constraint from negative or antagonistic pleiotropy is its effect on the number and fitness effects of beneficial mutations, and the associated probability that the same mutation will be repeatedly selected under the given conditions [6]. The chance that two independently evolving populations adapt by selecting the same beneficial mutation becomes relatively high when mutations are present with an exceptional benefit - as was recently found for the resistance to cefotaxime caused by mutations in TEM-1 β-lactamase [7].

Epistasis, the non-additive interaction of mutations at multiple loci, plays a major role when resistance to antibiotics requires the sequential substitution of multiple mutations. Sign epistasis - which occurs when mutations are beneficial in one background and deleterious in other backgrounds - introduces particularly strong constraints. It reduces the number of mutational pathways leading from sensitive to highly resistant mutants that are accessible to natural selection [8], thereby making evolution more predictable. Mutational pathways can be visualized on fitness landscapes, where fitness is mapped onto genotypes carrying all possible combinations of a set of mutations that contribute to adaptation. In theory, with full knowledge of the fitness landscape one should be able to predict the most likely path leading to full antibiotic resistance under any given condition; such predictions may be extended to other conditions with knowledge of the pleiotropic effects of the mutations (Figure 1b). Even for a single condition, full knowledge of the fitness landscape is, however, unrealistic, because the number of combinations grows exponentially with the number of contributing mutations. Current attempts to study empirical fitness landscapes by systematically analysing mutants carrying all possible combinations of sets of interesting mutations [9] are therefore necessarily restricted to sets of a handful of mutations. A complicating factor is that the predictability of evolution not only depends on knowledge of the fitness landscape, but also on population dynamic variables, such as population size and mutation rate, which determine which of the possible pathways will actually be followed [10].

The prospect of predicting the evolution of antibiotic resistance may seem utopic, but it is gaining momentum. One stimulus is the growing number of observations of the repeated fixation of the same small set of resistance mutations in independently evolving populations [9], to which the study of Rodríguez-Verdigo *et al*. [2] can now be added. A growing number of evolutionary biologists use antibiotic resistance as an experimental system. The information on the molecular changes involved in antibiotic resistance that will result from these endeavors will feed models of evolution that can incorporate observed patterns of pleiotropy and epistasis and explore the consequences under various population dynamic scenarios. With the aid of such models, we can start predicting what will happen when we expose pathogens to antibiotics, raising the prospect of devising strategies to reduce the probability that antibiotic resistance will arise. However, let us not be too optimistic: while we may be able to predict what happens when an antibiotic is present under controlled conditions in the laboratory, it will be even more challenging to predict how resistance develops under conditions that are unpredictable themselves.
